# The Philadelphia chromosome in leukemogenesis

**DOI:** 10.1186/s40880-016-0108-0

**Published:** 2016-05-27

**Authors:** Zhi-Jie Kang, Yu-Fei Liu, Ling-Zhi Xu, Zi-Jie Long, Dan Huang, Ya Yang, Bing Liu, Jiu-Xing Feng, Yu-Jia Pan, Jin-Song Yan, Quentin Liu

**Affiliations:** Department of Hematology, The Second Affiliated Hospital, Institute of Cancer Stem Cell, Dalian Medical University, Dalian, 116023 Liaoning P. R. China; State Key Laboratory of Oncology in South China, Sun Yat-sen University Cancer Center, Guangzhou, 510060 Guangdong P. R. China; Department of Oncology, The Second Affiliated Hospital, Dalian Medical University, Dalian, 116023 Liaoning P. R. China; Department of Hematology, The Third Affiliated Hospital, Sun Yat-sen University, Guangzhou, 510630 Guangdong P. R. China

**Keywords:** Chronic myeloid leukemia, BCR-ABL1, Philadelphia chromosome, Translocations, Signaling pathway

## Abstract

The truncated chromosome 22 that results from the reciprocal translocation t(9;22)(q34;q11) is known as the Philadelphia chromosome (Ph) and is a hallmark of chronic myeloid leukemia (CML). In leukemia cells, Ph not only impairs the physiological signaling pathways but also disrupts genomic stability. This aberrant fusion gene encodes the breakpoint cluster region-proto-oncogene tyrosine-protein kinase (BCR-ABL1) oncogenic protein with persistently enhanced tyrosine kinase activity. The kinase activity is responsible for maintaining proliferation, inhibiting differentiation, and conferring resistance to cell death. During the progression of CML from the chronic phase to the accelerated phase and then to the blast phase, the expression patterns of different BCR-ABL1 transcripts vary. Each BCR-ABL1 transcript is present in a distinct leukemia phenotype, which predicts both response to therapy and clinical outcome. Besides CML, the Ph is found in acute lymphoblastic leukemia, acute myeloid leukemia, and mixed-phenotype acute leukemia. Here, we provide an overview of the clinical presentation and cellular biology of different phenotypes of Ph-positive leukemia and highlight key findings regarding leukemogenesis.

## Background

Leukemia has among the highest mortalities of any cancer [1]. Chronic myeloid leukemia (CML) accounts for 15%–20% of all adult leukemias [2]. The Philadelphia chromosome (Ph) is the truncated chromosome 22 generated by the reciprocal translocation t(9;22)(q34;q11) and was first identified in 1960 in a patient with CML [3]. Translocation of the proto-oncogene tyrosine-protein kinase (*ABL1*) gene located on chromosome 9 to the breakpoint cluster region (*BCR*) gene located on chromosome 22 results in a *BCR*-*ABL1* fusion gene on the Ph [4, 5]. Three *BCR*-*ABL1* fusion gene hybrids encode BCR-ABL1 protein isoforms p210, p190, and p230, which have persistently enhanced tyrosine kinase (TK) activity. These aberrantly activated kinases disturb downstream signaling pathways, causing enhanced proliferation, differentiation arrest, and resistance to cell death [6, 7]. Tyrosine kinase inhibitors (TKIs) targeting the BCR-ABL1 protein are the most successful targeted therapy for Ph-positive leukemia. However, therapeutic resistance and disease progression are the current barriers to improve the prognosis of patients with Ph-positive leukemia [8–10]. Leukemia stem cells and BCR-ABL kinase domain mutations may be the keys to solve these problems [11]. The Ph is not limited to CML; it is also detected in cases of acute myeloid leukemia (AML) [12, 13], acute lymphoblastic leukemia (ALL; almost all of which are B-cell ALL, rarely T-cell ALL) [14], and mixed-phenotype acute leukemia (MPAL) [15–17]. The presence of the Ph results in patients with different leukemia phenotypes having substantially different prognoses. In addition, other concurrent genomic abnormalities are more common in leukemia cells with Ph than in those without. These genomic variations, in combination with BCR-ABL1 transcripts, play an important role during leukemogenesis [18–20].

However, the extent of the occurrence of the Ph and the types of *BCR*-*ABL1* transcripts found in different leukemia phenotypes, the exact role of the translocation in leukemogenesis, and the culprit of therapeutic resistance are still not fully elucidated. Here, we review the current understanding of this topic.

## The Ph, *BCR*-*ABL1* fusion gene, and BCR-ABL hybrid protein

**Fig. 1:**
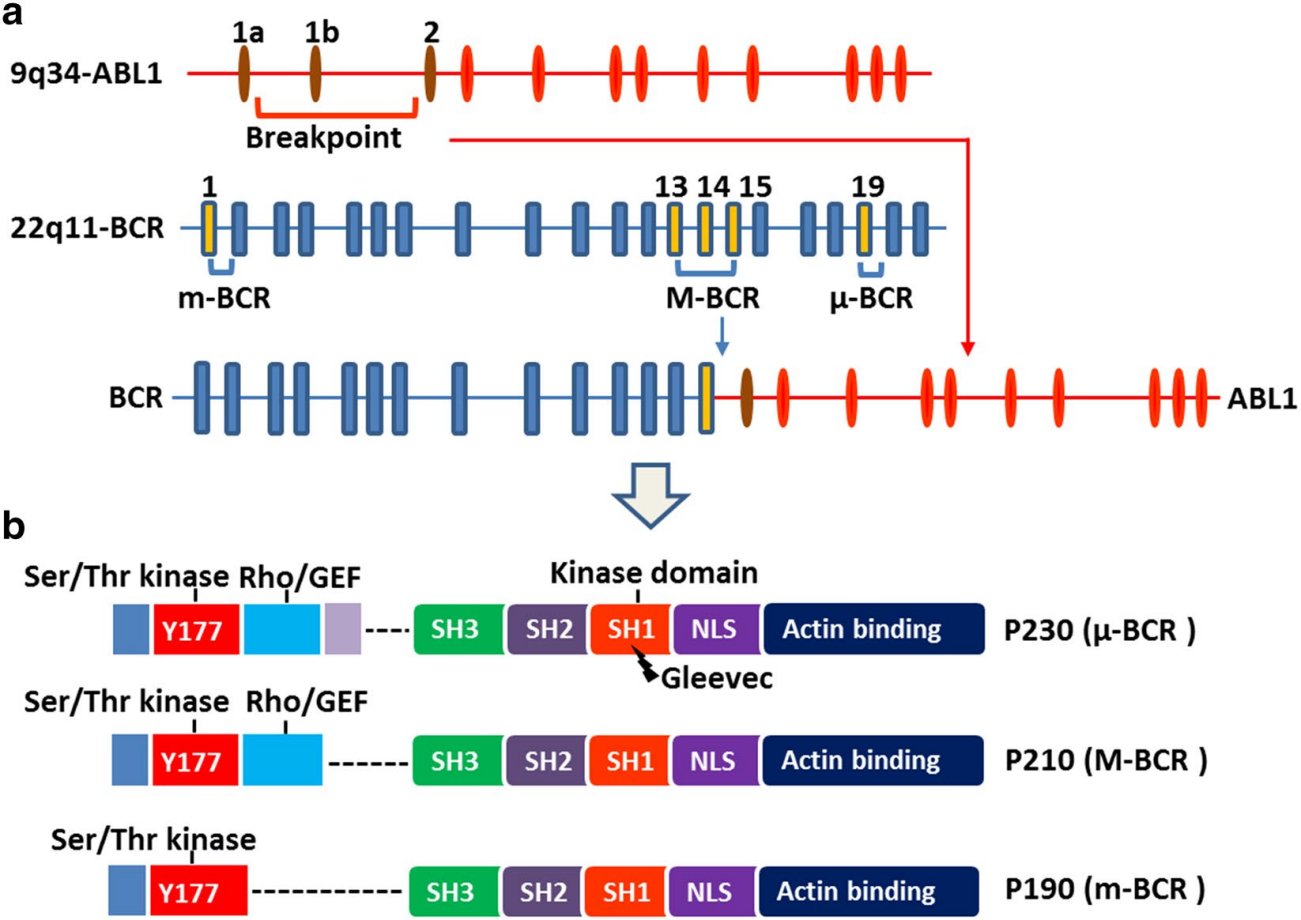
The structure of the breakpoint cluster region (*BCR*)-proto-oncogene tyrosine-protein kinase (*ABL1*) gene and protein. **a** The *BCR*-*ABL1* fusion gene consists of the 5′ end of the *BCR* gene located at 22q11 and the 3′ end of the *ABL1* gene located at 9q34. The breakpoints of the translocation usually involve the intron 13 or 14 of *BCR*, named major breakpoint (M-BCR), intron 1 of *BCR*, named minor breakpoint (m-BCR), and exon 19 of *BCR*, named μ breakpoint (μ-BCR). For *ABL1*, the breakpoint always involves the region between exons 1b and 2. **b** The BCR-ABL1 protein contains the Ser/Thr kinase domain and the Rho/GEF domain of BCR and the src homology (SH) domains, nuclear localization signal (NLS), and actin-binding domains from ABL1. The SH1 kinase domain is the target of imatinib. The different breakpoints that generate the p230, p210, and p190 isoforms are shown

Molecular investigation into the Ph observed in CML revealed a consistent genomic recombination between two genes—*BCR* on the long arm of chromosome 22 and *ABL1* on the long arm of chromosome 9—resulting in their juxtaposition, which generates the *BCR*-*ABL1* fusion gene [21]. The location of the *BCR* and *ABL1* genomic breakpoints is highly variable [22], but the recombination usually involves fusion of intron 1, intron 13/14, or exon 19 of *BCR* with a 140-kb region of *ABL1* between exons 1b and 2 (Fig. 1a). Referred to as p210^BCR-ABL1^, the fusion of *BCR* exon 13 and *ABL1* exon 2 (e13a2) or e14a2 constitutes the major *BCR*-*ABL1* transcript (M-BCR, originally referred to as b2a2 and b3a2). Both transcripts result in a hybrid 210-kDa protein. p210^BCR-ABL1^ is most commonly detected in CML and occasionally in ALL or AML. p190^BCR-ABL1^ (e1a2) constitutes the minor *BCR*-*ABL1* transcript (m-BCR), which encodes a hybrid 190-kDa protein. p190^BCR-ABL^ is commonly detected in B-cell ALL (B-ALL) and occasionally in AML but is rarely observed in CML [7]. p230^BCR-ABL1^ (e19a2), also known as the μ *BCR*-*ABL1* transcript (μ-BCR), encodes a hybrid 230-kDa protein. p230^BCR-ABL1^ is generated by the fusion of almost the entire *BCR* gene with the *ABL1* gene and is considered a molecular diagnostic marker for neutrophilic-chronic myeloid leukemia (CML-N) [23].

The BCR-ABL1 protein in CML contains several domains from both BCR and ABL1. The domains from BCR include an N-terminal coiled-coil domain (CC; amino acids 1–63), a Ser/Thr kinase domain containing a docking site (phosphorylated tyrosine 177, Y177) for the adaptor protein growth factor receptor-bound protein 2 (GRB2) [24, 25], and a ras homolog gene family/Guanine nucleotide exchange factors (Rho/GEF) kinase domain (amino acids 298–413) [26], whereas the domains from ABL1 include src homology (SH) domains (SH1/SH2), a proline-rich domain, and DNA- and actin-binding domains. Although different transcripts encode different proteins, a common feature of all the hybrid proteins (p210/190/230) is the constitutively active protein kinase activity compared to wild-type *ABL1* (Fig. 1b).

The N-terminal CC domain and Y177 of BCR are essential for the activation of ABL1 kinase [27, 28]. Targeting the CC domain to disrupt the tetramerization of BCR-ABL1 reduces its kinase activity and increases sensitivity to the TKI imatinib mesylate (imatinib, also known by the trade names Gleevec or Glivec) [29, 30], thus indicating that inhibition of tetramerization can contribute to overcoming imatinib resistance. In CML, Y177 plays a critical role in leukemic cell progenitor expansion, proliferation, and survival. Mutation of the GRB2-binding site at Y177 in p210^BCR-ABL1^ fails to induce a CML-like disease [24] and enhances sensitivity to imatinib by inhibiting RAS and protein kinase B (PKB, also named AKT) activation in CML [31]. These results show that Y177 is essential for transformation of CML by BCR-ABL1, and that it has potential as a target for overcoming imatinib resistance. The Rho/GEF protein plays a major role in activating differentiation in BCR-ABL1-induced leukemogenesis [32]. Inhibition of Rho kinase suppresses DNA synthesis in BCR-ABL1-transfected cells and also inhibits the proliferation and survival of CML progenitor cells [33, 34].

ABL1 protein, one of the non-receptor TKs, is present throughout hematopoietic development, with declining levels during myeloid maturation. The autophosphorylation site in the activation loop of its SH1 domain constitutes a switch between the inactive and active kinase conformation as it conjugates adenosine triphosphate (ATP) [35]. Imatinib and other kinase inhibitors compete with ATP to bind the autophosphorylation site, blocking the downstream signaling pathway [36]. In the “closed” conformation, the SH2 domain inhibits ABL1 activity, whereas in the “open” conformation, it promotes ABL1 activation through binding to the C-terminal of the SH1 domain [37]. Importantly, docking of the SH2 domain to the C-lobe of the kinase is controlled by the myristate moiety at the N-terminal of the SH3 domain. N-terminal myristoyl modification of the SH3 domain of ABL1 binds to the SH1 domain (the kinase domain) and induces conformational changes that allow the SH2 and SH3 domains to dock onto it [38, 39]. Mutant SH3 domain of ABL1 (exon 1b) with a blocked myristate-binding site shows strongly deregulated TK activity [38]. Also, blocking the myristate-binding site completely abolished leukemogenesis in mice and increased the sensitivity of imatinib-resistant BCR-ABL1-bearing mutants to TKI inhibition [40, 41]. These findings suggest that the myristate-binding site of the SH3 domain of ABL1 is a potential novel allosteric target for pharmacologic intervention.

## Ph in different phenotypes of leukemia

### Cml

The *BCR*-*ABL1* fusion gene is a hallmark of CML. Three discrete clinical stages are defined for CML: the chronic phase (CML-CP), the accelerated phase (CML-AP), and the blast crisis (CML-BC). Without therapeutic intervention, the disease follows a natural progression from relatively benign CML-CP, through CML-AP, to terminal CML-BC. The phenotype of CML-BC can be myeloid or lymphoid or, in rare cases, both. Myeloid BC is more commonly observed than lymphoid BC. In lymphoid BC, the predominant lineage is B-cell, representing about 30% of cases [42].

Most CML patients have M-BCR transcripts with b14a2 (55%) or b13a2 (40%) junctions (p210^BCR-ABL1^). In 5% of CML cases, both b13a2 and b14a2 transcripts are detected [43, 44]. However, the e1a2 (p190^BCR-ABL1^) transcript is frequently present at a low level in patients with p210^BCR-ABL1^-positive leukemia [45]. Approximately 52% of Ph-positive CML cases co-express p210^BCR-ABL1^ and p190^BCR-ABL1^ transcripts, with the other 48% exclusively expressing p210^BCR-ABL1^ [44]. All CML-BC patients co-express both transcripts [44]. Further details were provided in a study of 250 Mexican Ph-positive CML patients, which found that 90.4% of patients expressed p210^BCR-ABL1^, and approximately 7% of patients with p210^BCR-ABL1^ expressed both isoforms (b3a2/b2a2); however, co-expression of p190/p210^BCR-ABL1^ was seen in only 5% of patients [46]. Nevertheless, the prognosis for CML patients who co-express two or all p190/210/230^BCR-ABL1^ transcripts is poor [46]. Consistently, CML patients who co-express p210/p190^BCR-ABL1^ have considerably higher white blood cell (WBC) and blast cell counts at any time of testing, including diagnosis, than patients who express only p210^BCR-ABL1^ [44].

The position of the BCR breakpoint is also associated with prognosis. M-BCR rearrangement is predictive of response to therapy [47], whereas the presence of a double Ph indicates a poor prognosis [42]. However, no significant survival difference was found between patients with b13a2 and those with b14a2 mRNA junctions [48].

### Acute lymphoblastic leukemia

*BCR*-*ABL1* is not restricted to CML. It is also present in 11%–29% of ALL patients [49] but is relatively rare in childhood ALL (1%–3%) [50]. BCR-ABL1 presence in ALL patients increases with age [51] and has been reported to be as high as 50% in patients 60 years of age or older [52]. With few exceptions, Ph-positive ALL patients are diagnosed with B-ALL [53–55], and most cases of Ph-positive ALL express the p190^BCR-ABL1^ transcript. The p210^BCR-ABL1^ transcript is detected in 30% of adult and 20% of childhood patients with Ph-positive ALL [43, 45, 56–58]. The *BCR*-*ABL1* variant e3a2 (exon 3 of BCR and exon 2 of ABL1) can also be detected in cases of Ph-positive ALL, which is similar to ALL with p190^BCR-ABL1^ transcript [59].

Prognosis of both adults and children with Ph-positive ALL treated with standard chemotherapy is very poor, with less than 5% of adults being cured [55, 60]. Fortunately, the combination of chemotherapy with the TKI imatinib has remarkable efficacy on newly diagnosed Ph-positive ALL, achieving a complete remission rate of 95% and a patient survival rate of 55% at 3 years [61].

### Acute myeloid leukemia

*BCR*-*ABL1* transcripts are rarely found in AML. Less than 1.5% of AML patients harbor the *BCR*-*ABL1* fusion gene [62–64]. Ph-positive AML is cytogenetically indistinguishable from Ph-positive CML, but molecular studies show that, in 50% of cases, the breakpoint on chromosome 22 in Ph-positive AML is different from those very consistently found in CML [65]. Furthermore, studies have confirmed that *BCR*-*ABL1*-positive AML is a unique acute leukemia with some features distinct from myeloid CML-BC. These features include less marked splenomegaly, fewer peripheral basophiles, and a lower myeloid/erythroid ratio, and bone marrow cellularity [64] compared with myeloid CML-BC. In addition, the loss within the immunoglobulin genes (deletion of 14q32) in some cases of *de novo* Ph-positive AML can distinguish it from myeloid CML-BC [66]. However, the median survivals of Ph-positive AML and myeloid CML-BC patients are similar (9 vs. 7 months, *P* = 0.54) [64].

### Mixed-phenotype acute leukemia

The Ph is one of the most frequent aberrant cytogenetic findings in MPAL. The World Health Organization (WHO) defines MPAL as acute leukemia with a mixed phenotype containing two morphologically and immunophenotypically distinct populations of blasts or showing a single blast cell population expressing mixed phenotypic markers [67]. Many of these cases have a dimorphic population of blasts, with the majority being of B-lymphoid/myeloid lineage (59%–60%), followed by T-lymphoid/myeloid (32%–35%), T-/B-lymphoid (4%), and trilineage (2%–4%) [16, 68]. Ph-positive MPAL is defined as acute leukemia meeting the criteria for MPAL in which the blasts also have a detectable Ph or *BCR*-*ABL1* fusion transcript. The frequency of Ph-positive MPAL is 17%–35% in adult MPAL patients, whereas studies of pediatric patients report a much lower rate of 3% [69, 70]. No obvious difference was found between the *BCR*-*ABL1* transcript types expressed in adult cases [71, 72], but 30% of cases have additional chromosomal aberrations [72]. Importantly, compared with other phenotypes of leukemia, Ph-positive MPAL has a much worse outcome [16, 68]. Studies of Ph-positive MPAL are rare, and there is no consensus on the most appropriate therapy for this subtype. Although TKIs have improved the prognosis of Ph-positive MPAL, adult patients are still considered for hematopoietic stem cell (HSC) transplantation during the first remission.

## What role does Ph play in leukemogenesis?

The BCR-ABL1 fusion protein was first indicated as the crucial driver of CML in mouse studies, which showed that expression of p210^BCR-ABL1^ in the bone marrow caused a CML-like disease. The progression of p210^BCR-ABL1^-associated disease in transgenic mice is consistent with the apparent indolence of human CML-CP [73, 74]. Expression of p190^BCR-ABL1^ at a level similar to that in the p210^BCR-ABL1^-transgenic model results in a clinically distinct condition. Voncken et al. [75] demonstrated the development of de novo B-cell leukemia in mice exclusively transgenic for p190^BCR-ABL1^, with a relatively short period of latency. Furthermore, Castellanos et al. [76] created an in-frame fusion of p190^BCR-ABL1^ that mimics the human chromosomal translocation by homologous recombination in embryonic stem cells. The chimeric mice generated with the mutant embryonic stem cells systematically developed B-ALL, which was detected with elevated TK activity [77]. The TKI imatinib, the first agent targeting the TK activity of BCR-ABL1 protein, has become the first-line therapy for all patients with Ph-positive CML; it is also an indispensable therapy for Ph-positive ALL. Imatinib has changed the prognosis of CML radically over the last 15 years and improved the overall survival of ALL patients.

In addition to its TK activity, the SH2 domain of BCR-ABL1 is also required for induction of CML-like disease in mice, but, interestingly, SH2 is not required for lymphoid leukemogenesis [78]. BCR-ABL1 with deleted SH2 or the R1057K mutant on SH2 of p210^BCR-ABL1^ retains the ability to induce a fatal myeloproliferative disorder (MPD) with an extended latency [79]. Consistently, in cells transfected with SH2-mutated BCR-ABL1 or BCR-ABL1, the B-lymphoid expansion was diminished, suggesting that the BCR-ABL1-induced MPD suppresses B-lymphoid expansion [79].

### Pathways associated with BCR-ABL1

**Fig. 2:**
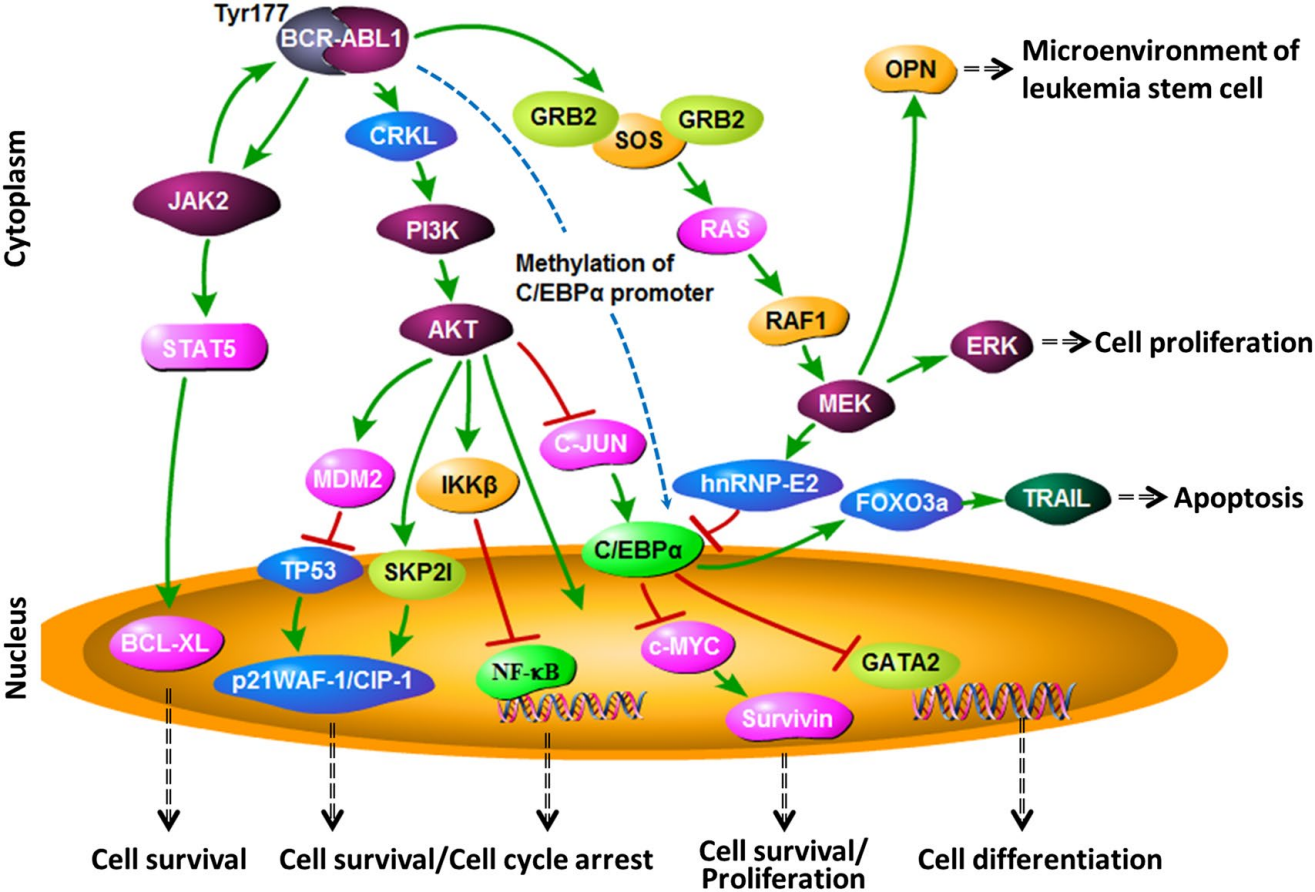
Main pathways regulated by the BCR-ABL1 protein. The downstream pathways regulated by BCR-ABL1 protein associated with cell survival, cell proliferation, cell cycle, cell differentiation, apoptosis, and the microenvironment of leukemia stem cells. *Dark violet* *shapes* indicate the kinase; *lavender pink shapes* indicate the proven oncoprotein; *blue shapes* indicate the proven tumor suppressor protein; and *other color shapes* indicate the proteins existing in normal cells. *Red solid lines* indicate inhibiting; *green solid lines* indicate activating. *Blue dotted lines* indicate that the results need further confirmation. *Black dotted lines* indicate the functions associated with the signaling pathway. *JAK2* janus kinase 2, *STAT5* signal transducers and activators of transcription 5, *BCR*-*XL* B-cell lymphoma-extra-large, *CRKL* Crk-like protein, *PI3* *K* phosphatidylinositol 3-kinase, *AKT* protein kinase B, *MDM2* mouse double minute 2 homolog, *TP53* tumor protein p53, *p21WAF*-*1/CIP*-*1* cytosolic cyclin-dependent kinase inhibitor p21, *SKP2I* S-phase kinase-associated protein 2 inhibitor, *NF*-*κB* nuclear factor kappa-light-chain-enhancer of activated B cells, *IKKβ* IKβ kinase, *C/EBPα* CCAAT/enhancer-binding protein α, *GATA2* GATA binding protein 2, *GRB2* the adaptor protein growth factor receptor-bound protein 2, *SOS* son of sevenless, *MEK* mitogen-activated protein kinase kinase, *ERK* extracellular signal-regulated kinases, *hnRNP*-*E2* poly(rC)-binding protein E2, *FOXO3a* forkhead box O3, *TRAIL* TNF-related apoptosis-inducing ligand

The transforming activity of BCR-ABL1 is due to its constitutive TK activity, which contributes to the maintenance of cell proliferation, inhibits differentiation, and promotes resistance to cell death. BCR-ABL1 kinase hyperactivity results in the activation of signaling pathways and deregulation of cellular processes [80]. Most of these pathways have been demonstrated in CML and ALL mouse models. The main pathways associated with BCR-ABL1 activity are presented in Fig. 2.

#### The JAK2/STAT pathway

Activation of Janus kinase (JAK)1–3 [81] and signal transducers and activators of transcription (STAT)1, 3, 5, and 6 [81, 82] has been confirmed experimentally in p190/p210^BCR-ABL1^-positive leukemia. JAK1–3 activation is mediated by the interaction of BCR-ABL1 with cytokine receptors [83]. BCR-ABL1 kinase directly enhances JAK2/STAT activation to promote cell growth/survival in CML models [84, 85], and it requires an intact JAK2/STAT5 pathway to enable oncogenic transformation [83]. JAK3 expression is limited to hematopoietic cells, and JAK3-knockout mice have developmental defects in lymphoid cells and expansion of the myeloid lineages [86–89]. JAK2 directly phosphorylates BCR-ABL1 at Y177 and increases BCR-ABL1 protein stability, thus enhancing BCR-ABL1 signaling [24, 90]. Moreover, JAK2 induces mRNA expression of the oncogene *c*-*MYC* and protects c-MYC protein from degradation [91]. c-MYC overexpression plays a critical role in BCR-ABL1 transformation [92] and is a downstream target of activated JAK2 in BCR-ABL1-positive cells. Survivin is overexpressed in BCR-ABL1-positive cells [93], and BCR-ABL1 activates c-MYC to induce transactivation of the survivin promoter via JAK2/phosphatidylinositol 3-kinase (PI3K) signaling pathways [94]. These findings indicate that JAK2 controls BCR-ABL1 stability and oncogenic signaling in BCR-ABL1-positive cells [95]. Both JAK2 inhibition and knockdown reduce levels of phosphorylated STAT5 (p-STAT5) and inhibit the RAS-PI3K pathway. By contrast, JAK2 inhibition decreases phosphorylated Y177 (p-Y177) but does not reduce levels of BCR-ABL1, suggesting that the reduction of BCR-ABL1 by JAK2 inhibition is a separate event from phosphorylation of Y177 [90]. A recent study showed that absence of JAK2 in a CML-CP model drastically accelerated disease development with increased numbers of WBC counts and severe splenomegaly, suggesting that JAK2 inhibits the progress of CML [96].

The level of p-STAT3 is higher in CML patients who are resistant to imatinib treatment than in patients who respond [97]. BCR-ABL1 regulates transcription of STAT3 by phosphorylating JAK1/2 and mitogen-activated protein kinase kinase (MEK) [98]. Both STAT3 and STAT5a/b are essential for the induction of CML-like leukemia by BCR-ABL1 and for the maintenance of the survival and growth of CML cells [95]. STAT5- or STAT3-knockout mice with BCR-ABL1 fail to display a CML phenotype and prevent established B-ALL. Additionally, STAT5, not STAT3, is essential for cell cycle progression and survival of lymphoid leukemic cells [95]. The N-terminal of STAT5a/b plays a key role in B-lymphoid transformation [99]. Furthermore, STAT5 is not essential for normal hematopoiesis [95, 100], which makes it a good therapeutic target in Ph-positive leukemia [84, 85].

#### The PI3K-AKT-mTOR pathway

The PI3K-AKT-mammalian target of rapamycin (mTOR) pathway is another important downstream cascade in Ph-positive leukemia, including CML and ALL. It is able to activate c-kit-positive HSCs from the quiescent to the proliferative state in BCR-ABL1-positive CML [101]. Through a PI3K-AKT-mTOR-independent pathway, BCR-ABL1 evades cell cycle arrest by increasing cytosolic cyclin-dependent kinase inhibitor p21 (p21WAF-1/CIP-1), which has been reported to have a pro-survival role localizing in cytoplasm [102]. BCR-ABL1 also induces S-phase kinase-associated protein 2 (Skp2) expression to promote proliferation of CML cells by the PI3K-AKT-mTOR pathway [103]. Meanwhile, BCR-ABL1 increases glucose metabolism and activates mitochondrial electron transport chains specifically regulated by PI3K-AKT-mTOR in CML cells [104, 105]. Ablation of PI3 K prevents BCR-ABL1 leukemogenesis in mice, and a dual PI3 K/mTOR inhibitor PI-103 suppresses proliferation of mouse pre-B-ALL more effectively than rapamycin [106]. The dual PI3 K and mTOR small-molecule inhibitors are also effective against TKI-resistant BCR-ABL1 mutant cells in vivo and in vitro [107]. In addition, inhibition of PI3K shows synergy with TKI-enhancing cytotoxic effects in p210^BCR-ABL1^-transformed cells, especially apoptosis [107, 108]. Taken together, these findings suggest that the PI3K-AKT-mTOR pathway plays an important role in BCR-ABL1-mediated leukemogenesis.

Currently, several PI3K inhibitors are being investigated, and several clinical trials have been completed in pediatric ALL [109]. Interestingly, a recent study showed that PI3K inhibitors induced global transcriptional reprogramming in tumors, with (re)phosphorylation of AKT and mTOR, and increased tumor cell motility and invasion [110]. That might explain why PI3K inhibitors were not more clinically efficacious in the last 10 years. Therefore, combining PI3K inhibitors with other therapeutic approaches may be a promising future therapeutic strategy.

#### The MAPK/ERK (RAS/RAF/MEK/ERK) pathway

The RAS/RAF/MEK/extracellular signal-regulated kinases (ERK) pathway is a central signal transduction pathway, which transmits signals from cell surface receptors to nuclear transcription factors. In BCR-ABL1-positive leukemia cells, activation of the RAS/RAF/MEK/ERK pathway results in uncontrolled proliferation [111–113]. BCR-ABL1 transduces proliferative signals partly through activation of RAS via GRB2/GRB2-associated binding protein 2 (GAB2) phosphorylation dependent on phosphorylation of Y177 of BCR [31, 114]. Disruption of RAS signaling attenuates development of BCR-ABL1-induced CML-like disease in mice, but most BCR-ABL1-positive mice with mutant *RAS* eventually develop pro-B-ALL. This indicates that RAS is a critical target of BCR-ABL1 in the pathogenesis of CML but not B-ALL [115].

Blockage of MEK/ERK elevates cytotoxicity of histone deacetylase inhibitors in BCR-ABL1-expressing cells resistant to the TKIs gefitinib or imatinib and leads to erythroid differentiation [116, 117]. BCR-ABL1 also activates B-RAF kinase, which is an effector molecule of the RAS-related protein RAP1 and a potent activator of the MEK/ERK/ELK-1 signaling pathway. Inhibition of RAP1 activation inhibits the BCR-ABL1-induced activation of ERK-1 [113].

Leukemic microenvironment plays a role in promoting and maintaining leukemic cell proliferation and survivability [118]. In addition to BCR-ABL1 kinase activity, CML stem cell survival depends on the continuous support of the hematopoietic niche [84]. Osteopontin (OPN), a component of the stem cell niche, is overexpressed in BCR-ABL1-expressing cells. BCR-ABL1 induces OPN overexpression by activating a signaling cascade involving RAS, RAF-1, and MAPK, indicating that BCR-ABL1 maintains the microenvironment for leukemia stem cells possibly through the RAS/RAF/MEK/ERK pathway [119].

Interestingly, oncogene addiction analysis shows that due to MEK-dependent negative feedback, JAK2 plays little or no role in signal transduction when BCR-ABL1 is active. After prolonged BCR-ABL1 inhibition (more than 8 h), negative feedback is relieved and JAK2 becomes critically important as a mediator of STAT5 phosphorylation in the downstream pathways [120]. Maintaining negative feedback with oncoprotein inhibition may, therefore, best facilitate the effects of target agents.

#### TRAIL-induced apoptosis

Tumor necrosis factor (TNF)-related apoptosis-inducing ligand-TNFSF10 (TRAIL), a death receptor ligand, is down-regulated in BCR-ABL1-positive leukemia [121–123]. In BCR-ABL1-positive cell lines and primary ALL cells, TRAIL induces death-inducing receptor (DR4 and DR5)-dependent apoptotic cell death [124]. Treatment with imatinib enhances TRAIL-induced apoptosis [125, 126]. Inducing TRAIL-mediated cell death also inhibits cancer cell proliferation and suppresses growth of CML xenografts [127].

These results suggest the potential use of recombinant TRAIL as a novel therapeutic agent; they also suggest the possible contribution of endogenously expressed TRAIL in immunotherapy against BCR-ABL1-positive leukemia.

#### C/EBPα-mediated differentiation

CCAAT/enhancer-binding proteins (C/EBPs) are transcription factors that regulate normal myelopoiesis as well as myeloid disorders. BCR-ABL1 suppresses *C/EBPα* mRNA levels via the induction of the mitogen-activated protein kinases-poly(rC)-binding protein E2 (MAPK-hnRNP-E2) pathway [128–130]. Disruption of C/EBPα blocks the transition from common myeloid to granulocyte-monocyte progenitor cells [131]. The failure of myeloid precursors to differentiate into mature granulocytes is a unique characteristic of clinical progression from CML-CP to CML-BC. C/EBPα expression is reduced in BCR-ABL1-expressing cell lines and CML-BC cells. Loss of miR-328 in CML-BC also reduces expression of C/EPBα [132]. Meanwhile, phosphorylation at serine 21 of the C/EBPα protein is associated with differentiation effects in K562 cells [133]. Furthermore, C/EBPα deregulation and neutrophilic differentiation by BCR-ABL1 are reversed by imatinib [134]. The gene profile shows that activation of C/EBPα induces the expression of cell cycle- and apoptosis-related genes and enhances imatinib-induced apoptosis of K562 cells [135]. Restoration of C/EBPα expression induces terminal granulocytic differentiation and inhibits proliferation of leukemia cells in vitro and in vivo [136–138]. Moreover, the effects of C/EBPα in p210^BCR-ABL1^-expressing cells depend partly on transcriptional repression of c-MYC and GATA-2 [139]. However, absence of C/EBPα results in loss of myeloid identity in transgenic mice with BCR-ABL1-induced CML-like disease and, interestingly, causes erythroleukemia instead, suggesting that CEBPα is essential for BCR-ABL1-positive CML [140].

Ectopic expression of C/EBPα in primary human BCR-ABL1-positive B-ALL cells induces macrophage-like cells, which are unable to establish disease in xenograft hosts [141]. Epigenetic studies show that aberrant methylation in the CpG island of the promoter region of *C/EBPα* is a common event in CML, suggesting that regulating methylation of *C/EBPα* could be a new therapeutic direction for treating Ph-positive leukemia [142].

#### Autophagy

Autophagy can be manipulated for a better therapy on AML by inducing cooperation with apoptosis and differentiation [143]. Also, autophagy presents a potential target in BCR-ABL1-positive leukemia, particularly in TKI-resistant types [144–146]. Disruption of autophagy is a new strategy to treat imatinib-resistant CML patients. Pharmacologic or genetic inhibition of the Hedgehog pathway can markedly induce autophagy in BCR-ABL1-positive CML cells. Inhibition of both Hedgehog pathway and autophagy may be a potent new strategy to overcome drug resistance in CML [147]. Importantly, BCR-ABL1 suppresses apoptosis as well as autophagy, resulting in low basal levels of autophagy in BCR-ABL1-transformed cells. Treatment of BCR-ABL1-positive cells in vitro with chemical inhibitors of autophagy or by deletion of the autophagy-related 3 (*Atg3*) gene induces cell death. In a cell transfer model in vivo, *Atg3* deletion also prevented BCR-ABL1-mediated leukemogenesis [148]. Moreover, the therapeutic drug arsenic trioxide (As_2_O_3_) targeted BCR-ABL1 for autophagy degradation via a p62/the gene encoding p62 (SQSTM1)-dependent mechanism, which is mediated localization of the oncoprotein to the autolysosomes [149].

In conclusion, BCR-ABL1-positive cells particularly depend on autophagy for leukemogenesis, and regulation of autophagy may be a therapeutic approach for BCR-ABL1-positive leukemia.

### Genomic instability

Genomic instability is an essential factor of tumorigenesis for both leukemias and solid tumors. Chromosomal translocations generate the aberrant fusion TKs, such as BCR-ABL1, Ets variant gene 6-ABL1 [TEL(ETV6)/ABL1], TEL(ETV6)/JAK2, and TEL-platelet-derived growth factor beta receptor [TEL(ETV6)/PDGFβR], which induce hematologic malignancies [150]. BCR-ABL1-positive cells contain elevated numbers of DNA double-strand breaks (DSBs) and show stimulation of the single-strand annealing (SSA) repair process [151–153]. The *WRN* gene (mutated in Werner syndrome) encodes a helicase required for processing DSB ends during the repair. BCR-ABL1 enhances the expression and increases the nuclear localization of *WRN* to promote survival and genomic instability [154].

Prolonged expression of the p210^BCR-ABL1^ transcript was associated with development of aneuploidy and complex chromosomal translocations in the mouse model [155]. Transgenic mice expressing p190^BCR-ABL1^ developed karyotypic abnormalities, most commonly trisomies involving chromosomes 12, 14, or 17, alone or in combination [18].

A recent study using a whole transcriptome array showed that genes such as dipeptidyl peptidase-4 (*DPP4*), interleukin-2 receptor alpha (*IL2RA*), protein tyrosine phosphatase, receptor type D (*PTPRD*), calcium channel, voltage-dependent, L type, alpha 1D subunit (*CACNA1D*), interleukin 1 receptor accessory protein (*IL1RAP*), solute carrier family 4 (*SLC4A4*), and potassium channel, subfamily K, member 5 (*KCNK5*) were up-regulated in BCR-ABL1-positive CML. This study found that these genes play key roles in proliferation, differentiation, and molecular pathways in HSCs [156]. Accumulation of additional cytogenetic and molecular abnormalities also contributes to blast transformation and progression [157–159]. BCR-ABL1 and genomic instability are currently considered to be a complex partnership in leukemogenesis, suggesting that BCR-ABL1 itself leads to genomic instability independent of its leukemogenic effect [20].

The Ph rarely appears with other genetic abnormalities in CML-CP. However, in Ph-positive B-ALL, AML, MPAL, and CML-AP and CML-BP, deletions involving immunoglobulin heavy chain (*IGH*), T-cell receptor (*TCR*), encoding the transcription factor IKAROS family zinc finger protein 1 (*IKZF1*), and cyclin-dependent kinase inhibitor 2 (*CDKN2A/B*) are common [66]. A recent study showed that the *BCR*-*ABL1* fusion gene could be a prenatal and possibly initiating genetic event in Ph-positive childhood ALL and that variation in other genes is a secondary and probably postnatal event in these cases [160]. Common gene abnormalities found in Ph-positive leukemia are discussed in Table 1.

**Table 1:** Recurrent genomic abnormalities in Philadelphia chromosome (Ph)-positive leukemia

Genomic abnormality	Location	Status	Ph-positive leukemia	Frequency	Reference(s)
*IKZF1*	7q12.2	Deletion	ALL	50%–83%	[162, 164]
		Deletion	Lymphoid CML-BC	73%–75%	[163, 180]
*PAX5*	9p13	Deletion	ALL	33%–51%	[171, 172, 180]
		Deletion	Lymphoid CML-BC	58.3%	[180]
*EBF1*	5q34	Deletion	ALL	14%	[198]
*CDKN2A/B*	9p13-p23.1	Deletion	AML	50%	[66]
		Deletion	MPAL	33.3%	[66]
		Deletion	ALL	53.5%	[163, 164]
		Deletion	Lymphoid CML-BC	58.3%–69%	[180]
*IG*	14q32.33	*IGHV/IGHG2* *M* deletion	Lymphoid CML-BC	66%–100%	[66, 163, 180]
	22q11.2	*IGLL1* deletion	AML	66.7%	[66]
*TCR*	14q11.2/7p14.1	*TCRA/B/D* deletion	AML/MPAL	66.7%	[66]
	14q11.2	*TCRA* deletion	Lymphoid CML-BC	74%	[66, 180]
*BTG1*	12q21.33	Deletion	BCP-ALL	11%–31.3%	[176]
		Deletion	MPAL	33.3%	[176]
		Deletion	CML-BC (B-lineage)	33.3%	[176]

*IKZF1* transcription factor IKAROS family zinc finger protein 1, *PAX5* paired box 5, *EBF1* early B-cell factor 1, *CDKN2A/B* cyclin-dependent kinase inhibitor 2, *IG* immunoglobulin, *TCR* T-cell receptor, *BTG1* B-cell translocation gene 1, *IGHV* immunoglobulin heavy chain variable region, *IGHG2M* immunoglobulin heavy constant gamma 2, *IGLL1* immunoglobulin lambda-like polypeptide 1, *TCRA* T-cell receptor alpha locus, *ALL* acute lymphoblastic leukemia, *CML*-*BC* chronic myeloid leukemia blast crisis, *AML* acute myeloid leukemia, *MPAL* mixed-phenotype acute leukemia, *BCP*-*ALL* B-cell precursor ALL

#### *IKZF1* mutation

*IKZF1*, located on chromosome 7p12, is a regulator of lymphocyte differentiation. Wild-type IKAROS prevents stemness properties and has tumor suppressor activity in BCR-ABL1-initiated leukemia [161]. *BCR*-*ABL1* and *IKZF1* mutations are strongly linked: somatic mutations in *IKZF1* are present in 70%–83% of Ph-positive ALL cases; approximately 90% are deletions, and 10% are point mutations [162]. Deletion of *IKZF1* has also been identified as an acquired lesion at transformation from CML-CP to lymphoid CML-BC but never in myeloid CML-BC or AML with Ph [163]. In addition, loss of *IKZF1* predicts a poor prognosis in patients with Ph-positive leukemia [164]. Disruption of IKAROS activity in primitive CML-CP cells can mimic myeloid disease progression (CML-AP), revealing that loss of *IKAROS* is a frequent step and potential predictor of BCR-ABL1-positive CML-AP/BC [162]. The IKAROS-6 (IK6) is produced by an in-frame deletion of exons 4–7 of *IKZF1*, which deletes the DNA-binding domain and leads to cytosolic accumulation of the mutant protein. The mutations associated with a more profound reduction in IKAROS function (bi-allelic deletion and IK6) are particularly common in Ph-positive ALL [165].

In addition, loss of *IKZF1* is recurrent in pediatric AML and may be a determinant of oncogenesis in AML with monosomy 7 [166]. Sequencing of *IKZF1* deletion breakpoints suggests that aberrant recombination activating gene (RAG)-mediated V(D)J recombination is responsible for the deletions [167].

#### *PAX5* and *EBF1* mutation

The paired box 5 (PAX5) and early B-cell factor 1 (EBF1) are transcription factors that are expressed specifically during B-cell development and control lineage identity and commitment [168–170]. Recurrent deletions of *PAX5* and *EBF1* occur in approximately 50% and 14% of Ph-positive ALL cases, respectively [163, 168, 171]. In contrast to BCR-ABL1-negative ALL, no point mutations of *PAX5* have been found, suggesting that deletions are the main mechanism of inactivation of *PAX5* in BCR-ABL1-positive ALL [172]. *EBF1* co-regulates target genes with *PAX5*. In mouse models, loss of *PAX5* or *EBF1* leads to a differentiation block at the pro- to pre-B-cell stage, resulting in B-cell precursor leukemia (BCP-ALL) [170, 173]. Complete loss of *PAX5* and *EBF1* is apparently a secondary event and is significantly associated with *BCR*-*ABL1* [171].

#### *BTG1* deletion

The B-cell translocation gene 1 (*BTG1*) on chromosome 12q21.33 is highly conserved and belongs to an antiproliferative gene family that regulates cell growth, differentiation, and angiogenesis [174, 175]. *BTG1* deletion occurs in 11%–31.3% of BCP-ALL cases, in 33.3% of MPAL cases, and in 33.3% of CML-BC (B-lineage) cases [176]. Eight distinct deletions of different sizes within the second exon of *BTG1* have been identified in BCP-ALL, resulting in the expression of truncated *BTG1* transcripts [177]. Loss of *BTG1* expression also causes glucocorticoid (GC) resistance both by reducing glucocorticoid receptor (GR) expression and by controlling GR-mediated transcription in ALL [178]. It suggests that *BTG1* deletions may act as “drivers” of leukemogenesis in BCP-ALL, MPAL, and lymphoid CML-BC (B-lineage) with Ph. BTG1 overexpression inhibits proliferation and invasion and induces G_2_/M arrest, differentiation, senescence, and apoptosis in xenograft models of gastric cancer. Restoring BTG1 might reverse phenotypes and be a potential target for gene therapy of Ph-positive leukemia [179].

#### *IGH* and *TCR* deletion

The deletions of 4 chromosomal regions, 7p12–14, 9p21–24, 14q11.2, and 14q32.33, are found recurrently in Ph-positive leukemia. The deletions of immunoglobulin heavy chain (*IgH*) genes and *TCRα/β/γ* genes are restricted to the four chromosomal regions. Most common deletions include immunoglobulin heavy chain variable region (*IGHV*), immunoglobulin heavy constant gamma 2 (*IGHG2* *M*), and T-cell receptor alpha locus (*TCRA*). Deletion within both *IGH* and *TCR* genes is a characteristic of lymphoid CML-BC with Ph; none of these genome losses is detected in CML-CP or myeloid CML-BC samples [180]. Deletions within chromosomes 7 and 9, including *IKZF1* and *CDKN2a/b* genes, are also frequently accompanied by *IGH* and *TCR* gene deletion in Ph-positive BP-ALL [66], indicating that deletion of the *IGH* and/or *TCR* gene region is obligatory for the development of a malignant clone with a lymphoid phenotype. Interestingly, Ph-positive AML also possesses deletions of immunoglobulin (*IG*) and *TCR* genes but involving immunoglobulin lambda-like polypeptide 1 (*IGLL1*, 22q11.2) and *TCRA/B/D* (14q11.2 and 7p14.1), respectively. The above studies suggested that different parts of the deletion with *IG* and *TCR* genes might result in different phenotypes of leukemia [19, 66, 163].

## Therapeutic resistance and disease progression in Ph-positive leukemia patients

Compared with CML-CP patients, imatinib responses are much less durable in patients with CML-AP/BC or ALL [8–10]. Compelling research suggests that a population of cancer stem cells (CSCs) is responsible for therapeutic resistance and disease progression. BCR-ABL1-positive stem cells persist in CML patients despite prolonged treatment with imatinib [181, 182]. Primitive, quiescent, Ph-positive stem cells (CD34^+^CD38^–^) from patients with CML are insensitive to imatinib in vitro [183]. Consistently, the BCR-ABL1 expression in persistent leukemic stem cells could explain innate resistance to imatinib and to other TKIs [184].

In CML patients with TKI resistance, relapse or evolving to blast crisis, BCR-ABL1 kinase mutations (M237I, L248V, Q252E, Y253H, D276G, G321E, V304A, M351T, T315 I, E352G, Y353G, E373G, and T389A) can be detected in stem cells [185, 186]. Mutations were found in 27% of CML-CP patients, 52% of CML-AP patients, 75% of myeloid blast crisis patients, and 83% of lymphoid blast crisis/Ph-positive ALL patients [187]. Thirty percent of patients with primary resistance and 57% of patients with acquired resistance were associated with these mutations [187]. Studies have confirmed that the mutations were the potential source of resistance and relapse [185]. The following mutations are involved in theraputic resistence and disease progression [188]: (1) contact residues (such as T315) by impeding inhibitor access or eliminating critical hydrogen bonds; (2) the ATP-binding loop (such as L248V) by preventing Abl from adopting the specific conformation required for high-affinity imatinib binding; and (3) regulatory motifs (such as the activation loop) by stabilizing an active conformation that is inaccessible to imatinib.

Recent studies showed that inhibition of heat shock protein 90 (Hsp90) decreased the number of leukemia stem cells, caused BCR-ABL protein degradation by the ubiquitin-proteasome pathway, and prolonged survival of mice with CML induced by BCR-ABL-T315I [189, 190]. Many novel Hsp90 inhibitors have entered into clinical trials, the results of which are encouraging [190]. This anti-Hsp90 strategy in treating CML patients, especially with TKI resistance, has a good application prospect. Another study showed that some alkyne-containing pyrazolopyrimidines can inhibit not only Abl(T315I) in vitro but also Bcr-Abl(T315I) in cells, suggesting that these pyrazolopyrimidines can serve as lead compounds for targeted therapy to overcome drug resistance of CML [191]. Also, one third-generation TKI, ponatinib, has been proven against the drug resistance including the T315I mutation. X-ray crystallographic analysis revealed that ponatinib inactivated T315I Bcr-Abl mutated kinase by conformational alteration [192].

In brief, a quiescent population of leukemia stem cells with or without *BCR*-*ABL1* kinase domain mutations is responsible for drug resistance [11].

## Conclusion leukemogenesis is an outcome of the Ph combined with other genetic variations

Expression of *BCR*-*ABL1* (p210 transcript) has been detected at very low levels in the peripheral blood cells of some healthy individuals but not in umbilical cord blood cells [193]. In addition, *BCR*-*ABL1*-specific T cells are detected in healthy donors and in CML patients after allogeneic stem cell transplantation [194]. These results indicate that normal cells evolve progressively to a neoplastic state, and they may acquire a succession of genetic abnormalities and gain the ability to maintain proliferation, inhibit differentiation, and resist cell death [195].

The Ph bearing the *BCR*-*ABL1* fusion gene is the key initiator of different phenotypes of leukemia with diverse prognoses. The translocation leads to persistent TK activation and genomic instability during leukemogenesis. Disorders in multiple signaling pathways and genetic abnormalities combined with the Ph are essential for the evolution of different types of leukemia; however, why cells possessing the Ph should evolve specifically into CML, AML, ALL, or MPAL is currently unclear and under investigation. Evidence shows that there are characteristics exclusive to specific leukemias, including deletion of *BTG1* in B-cell leukemia, loss of *IKZF1* with monosomy 7 in AML, and deletions involving *IGH*, *TCR*, *IKZF1*, and *CDKN2A/B* in CML-AP/CP. Greater understanding of leukemogenesis and the effect of treatment on clonal evolution will provide novel insights into the design of future therapeutic strategies for Ph-positive leukemia [196, 197].

## Abbreviations

Ph: Philadelphia chromosome
CML: chronic myeloid leukemia
ALL: acute lymphoblastic leukemia
AML: acute myeloid leukemia
MPAL: mixed-phenotype acute leukemia
TK: tyrosine kinase
TKIs: tyrosine kinase inhibitors
CML-N: neutrophilic-chronic myeloid leukemia
Y177: phosphorylated tyrosine 177
CML-CP: chronic phase of CML
CML-AP: accelerated phase of CML
CML-BC: blast crisis of CML
myeloid CML-BC: myeloid-BC of CML
HSC: hematopoietic stem cell
MPD: myeloproliferative disorder
OPN: osteopontin
TRAIL: tumor necrosis factor-related apoptosis-inducing ligand-TNFSF10
C/EBPs: CCAAT/enhancer-binding proteins
SSA: single-strand annealing
IKZF1: encoding the transcription factor IKAROS
BTG1: B cell translocation gene 1
IGH: immunoglobulin heavy chain
EBF1: early B-cell factor 1
PAX5: paired box 5
CDKN2A/B: cyclin-dependent kinase inhibitor 2
TCR: T-cell receptor
IGHV: immunoglobulin heavy chain variable region
IGHG2M: immunoglobulin heavy constant gamma 2
TCRA: T-cell receptor alpha locus
IGLL1: immunoglobulin lambda-like polypeptide 1
